# Smooth pursuit and memory saccades are impaired in early-stage Parkinson’s disease patients

**DOI:** 10.3389/fneur.2025.1702050

**Published:** 2026-01-22

**Authors:** Zvonimir Popovic, Tihana Gilman Kuric, Ines Rajkovaca Latic, Sara Matosa, Luka Kusic, Andrea de Gobbis, Aleksander Sadikov, Vida Groznik, Dejan Georgiev, Svetlana Tomic

**Affiliations:** 1 Faculty of Medicine Osijek, Josip Juraj Strossmayer University of Osijek, Osijek, Croatia; 2 Department of Neurology, University Hospital Center Osijek, Osijek, Croatia; 3 Department of Gastroenterology and Endocrinology, Dr. Josip Bencevic General Hospital, Slavonski Brod, Croatia; 4 Faculty of Computer and Information Science, University of Ljubljana, Ljubljana, Slovenia; 5 Department of Neurology, University Medical Centre Ljubljana, Ljubljana, Slovenia

**Keywords:** biomarker, early-stage, eye movements, Parkinson’s disease, smooth pursuit

## Abstract

**Introduction:**

Parkinson’s disease (PD) is a progressive neurodegenerative disease caused by degeneration of dopaminergic neurons in substantia nigra pars compacta (SNc). One of the most prevalent symptoms is eye movement impairment, presenting in 75% of PD patients, which have fragmented and hypometric smooth pursuit movements with prolonged latency. We aimed to investigate differences in smooth pursuit, reflexive, and memory-guided saccades and antisaccades between patients with early-stage PD and healthy controls.

**Methods:**

We conducted a cross-sectional study with idiopathic PD patients in early stage of disease (Hoehn and Yahr stage 0, 1 and 2) and healthy controls. The impairment of smooth pursuit, saccades, antisaccades, and memory-guided saccades was evaluated with eye-tracker analysis using a battery of tests.

**Results:**

Forty-two subjects with early-stage idiopathic PD and 50 healthy controls participated in the study. There were no statistically significant differences in age, gender, years of education, or cognition between the groups. Early-stage PD patients showed impairment in velocity, phase, and range of motion of smooth pursuit eye movements, as well as impaired precision and recollection performing visually guided memory saccades. Consequently, there is also a reading dysfunction, with slower reading speed and longer eye fixations. No significant differences were found regarding reflexive saccades and antisaccades between these two groups.

**Conclusion:**

Results suggest that impaired smooth pursuit movements, memory-guided saccades and reading functions are present in early-stage PD, even without other expressed motor symptoms. These findings could potentially contribute to the development of new and non-invasive diagnostic biomarkers in PD.

## 1 Introduction

Parkinson’s disease (PD) is a progressive neurodegenerative disease caused by degeneration of dopaminergic neurons in substantia nigra pars compacta (SNc), which results in a heterogenous clinical picture. Although it is diagnosed clinically with the presence of characteristic motor symptoms (bradykinesia tremor, and rigidity) (1), eye movement impairment is often neglected. Nakamura et al. (2) found a prevalence of eye movement impairment of around 75%, while in the general population it is significantly lower and ranges from 31% in the age group 50 to 59 years to 51% in subjects older than 80 years (3).

Different types of eye movements are regulated with various neuroanatomical loops. Several studies using eye-trackers have shown that PD patients have fragmented and hypometric smooth pursuit movements with prolonged latency. Also, the horizontal and vertical saccades are slower and hypometric, with impaired precision (4–8). Research by Zhang et al. (9) has also detected changes in fixation stability and visual range during smooth pursuit eye movements. Furthermore, antisaccades in PD patients also have longer latency and a higher number of errors, possibly also contributing to dysexecutive syndrome in these patients (10). Neurodegeneration in PD affects also memory-guided saccades, which have prolonged latency, especially when integration of memory and visual information is needed (11). Patients with PD have trouble with initiation and execution of these saccades (12), which correlates with their cognitive state (13). The integration of different types of eye-movements in complex tasks, such as during reading, is also impaired in PD. For example, during reading, patients with PD present with hypometric saccades and fragmented smooth pursuit, which results in an increased number of corrective saccades (14). Similar to memory-guided saccades, cognitive function is invaluable in the integration of eye movements during reading (15).

What is still debatable is when the impairemet of different types of eye movements occur and whether they are present in the early stage of PD. The Hoehn and Yahr (H&Y) scale is a widely utilized tool for staging the progression of PD, categorizing patients based on the severity of their motor symptoms. In the early stages of PD (H&Y stages 0, 1 and 2), patients exhibit unilateral or bilateral motor symptoms without significant postural instability (16). Zhou et al. (17) have determined that there are already changes in saccadic latency and accuracy in *de novo* PD patients. According to already mentioned research by Terao et al. (6), reflexive saccades remain preserved until several years after disease onset. However, a newer study by Yu et al. (13) has shown that there is early impairment of reflexive saccades in PD.

We aimed to explore changes of smooth pursuit, reflexive, and memory-guided saccades and antisaccades in early-stage PD as compared to age and sex matched healthy controls. We used eye-tracker to objectively evaluate eye-movements. We hypothesised that patients in early-stage of disease would already have significantly impaired eye-movements.

## 2 Methods

### 2.1 Participants

This cross-sectional study was undertaken at the Department of Neurology, University Hospital Center Osijek. Detailed study design can be seen as Supplementary Figure 1. The diagnosis of idiopathic PD was established according to the clinical diagnostic criteria set forth by the Movement Disorder Society (18). PD patients with H&Y stages 0, 1, and 2 were included in the study (19, 20). The exclusion criteria included structural brain damage, uncorrected visual impairment including strabismus, amaurosis, amblyopia, or visual field defects that might have influenced eye movement, and dementia, as determined by the Movement Disorder Society’s clinical diagnostic criteria for dementia related to PD, alongside a Montreal Cognitive Assessment (MoCA) score of less than 18 (21).

### 2.2 Methods

Examinees filled out a standardized questionnaire on the age of disease onset, disease duration, dominant hand, disease lateralization, education, comorbidities, and PD treatment. Motor symptoms and disease stage were assessed using Movement Disorder Society Unified Parkinson Disease Rating Scale Part III and IV (MDS-UPDRS III and IV), while MoCA was used to assess cognition (22).

### 2.3 Eye movement recording and analysis

Eye movements were recorded and analysed by Tobii Eye Tracker 4C, which captures eye movements based on screen coordinates at a frequency of 90 Hz. Each stimulus was displayed as white dots against a black background (23). Prior to assessment onset, each patient was apprised of the anticipated procedures during the testing and thereafter underwent eye movement calibration. The test lasted for 10 to 15 min. All assessments were conducted ON medication.

The test battery consisted of tests for smooth pursuit, reflexive and memory saccades, as well as anti-saccades. Integration of eye movements was tested during reading. Data was recorded using software program Neus v2.0.

Smooth pursuit eye movements were evaluated by tracking a dot along the horizontal and vertical axes at three distinct velocities, defined by the duration of a single movement cycle. The quickest cycle lasted for 1,600 milliseconds (ms), the moderate cycle 2,400 ms, and the slowest cycle 4,800 ms (Supplementary Figure 2A). Each cycle and axis was defined by the accuracy, velocity, phase (distance to the stimulus during pursuit), and range of ocular movements, as well as the number of fixations. Accuracy, velocity, and phase were quantified using mean square error (MSE), with values ranging from 0 to 1, where values approaching 1 indicate a greater divergence from the stimulus.

Reflexive saccades were evaluated by instructing the participant to focus on the dot upon its appearance on the screen in both horizontal and vertical orientations (Supplementary Figure 2B). The latency of the initial accurate gaze and the actual latency (when a minimum of five accurate gazes were recorded) were assessed. For the antisaccades, we instructed participants to gaze in the opposite direction of the appearing dot, in a horizontal axis (Supplementary Figure 2C). We documented the number of corrections and accurate movements, the proportion of erroneous eye movements, actual latency, and the eye movement deviation.

Visually guided memory saccades were evaluated utilizing the Corsi test. The Corsi blocks were emphasized in a random sequence using distinct colors, and the test subject was required to recall and replicate this sequence. In a subsequent assessment, the participant was required to memorize the highlighted Corsi blocks and then indicate them in reverse sequence (Supplementary Figure 2D). The quantity of indicated blocks commenced at two and augmented with each accurate response. If an incorrect response was provided, the remainder of the Corsi test was terminated. Four Corsi trials (two in the forward direction and two in the backward direction) were conducted during the eye movement assessments. The analysis encompassed the quantity of accurate forward and backward hits, the number of correct hits in one trial, the peak level reached in one trial, the number of correct hits attained in a test, and the greatest overall level reached.

Additionally, we conducted a reading test, by asking the examinees to read a semantically meaningful text in their native (Croatian) language that required their attention and concentration. During reading, we recorded the percentage of text read (as a marker of average reading speed), percentage of text read before they return back to previous text, saccade velocity, ratio of number of saccades during reading forwards and backwards, the minimal and maximal number of eye fixations in 1 s, the duration of eye fixation, and the difference in the duration of different eye fixations.

### 2.4 Statistical analysis

The statistical analysis was conducted using SPSS 23.0 (IBM, Chicago, IL). We employed the Shapiro–Wilk test to assess the normality of the distribution of numerical data. Nominal variables were analysed using the Pearson chi-square test. We used the Student *t*-test to compare parametric and Mann–Whitney *U* test to compare non-parametric data. The statistical significance threshold was established at alpha = 0.05.

## 3 Results

### 3.1 Characteristics of examinees

Forty-two subjects with early-stage idiopathic PD and 50 healthy controls participated in the study. As can be seen in Table 1, there were no statistically significant differences in age, gender, years of education, or cognitive status between groups.

**Table 1:** Comparison of demographic parameters between patients with early-stage PD and healthy controls.

Variable		Examinees		*p*
		Early-stage PD	Healthy controls	
Sex	Male	32 (76.2%)	30 (60%)	0.09^†^
	Female	10 (23.8%)	20 (40%)	
Age (years)		66.5 (59.75–71)	63 (56.5–68)	0.07^*^
Duration of education (years)		12 (12–15.75)	12 (12–16)	0.23^*^
MoCA		27 (25–28.25)	27 (26.5–28)	0.34^*^

^†^Pearson’s chi-squared test.^*^Student’s t-test.PD, Parkinson’s disease; MoCA, Montreal Cognitive Assessment.

Regarding patients with early-stage PD, their age at disease diagnosis was 60 (53.75–65.25) years, and the average duration of disease was 4 (2–7) years, with an average levodopa-equivalent daily dose (LEDD; calculated using standard conversion formula) of 500 (400–772) milligrams. There were 10 (23.8%) patients in H&Y stage 0, 13 (31%) in stage 1, and 19 (45.2%) in stage 2, respectively. When observing their motor symptoms, the average MDS-UPDRS III score was 11 (9–15.25), while MDS-UPDRS IV score was 0 (0–0).

### 3.2 Differences in eye movements

**Figure 1:**
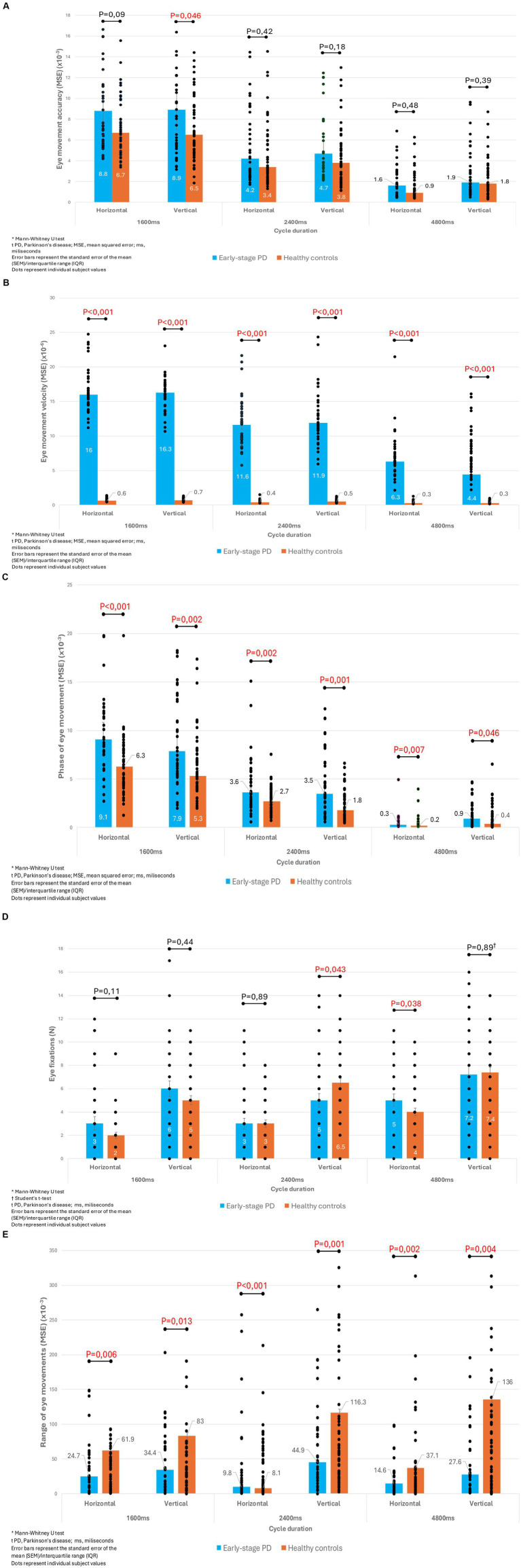
**(A)** Comparison of eye movement accuracy in all observed cycles between patients with early-stage PD and healthy controls. **(B)** Comparison of eye movement velocity in all observed cycles between patients with early-stage PD and healthy controls. **(C)** Comparison of phase of eye movement in all observed cycles between patients with early-stage PD and healthy controls. **(D)**. Comparison of number of eye fixations in all observed cycles between patients with early-stage PD and healthy controls. **(E)** Comparison of range of eye movements in all observed cycles between patients with early-stage PD and healthy controls.

We also compared the differences in the parameters of smooth pursuit movements between these groups of subjects. In all observed cycles, patients with early-stage PD had significantly slower velocity, a larger phase, and a smaller range of movement, both in the horizontal and vertical axes. Their accuracy was also impaired, but only in the fastest cycle in the vertical axis. These results can be seen in Figures 1A–E.

**Figure 2:**
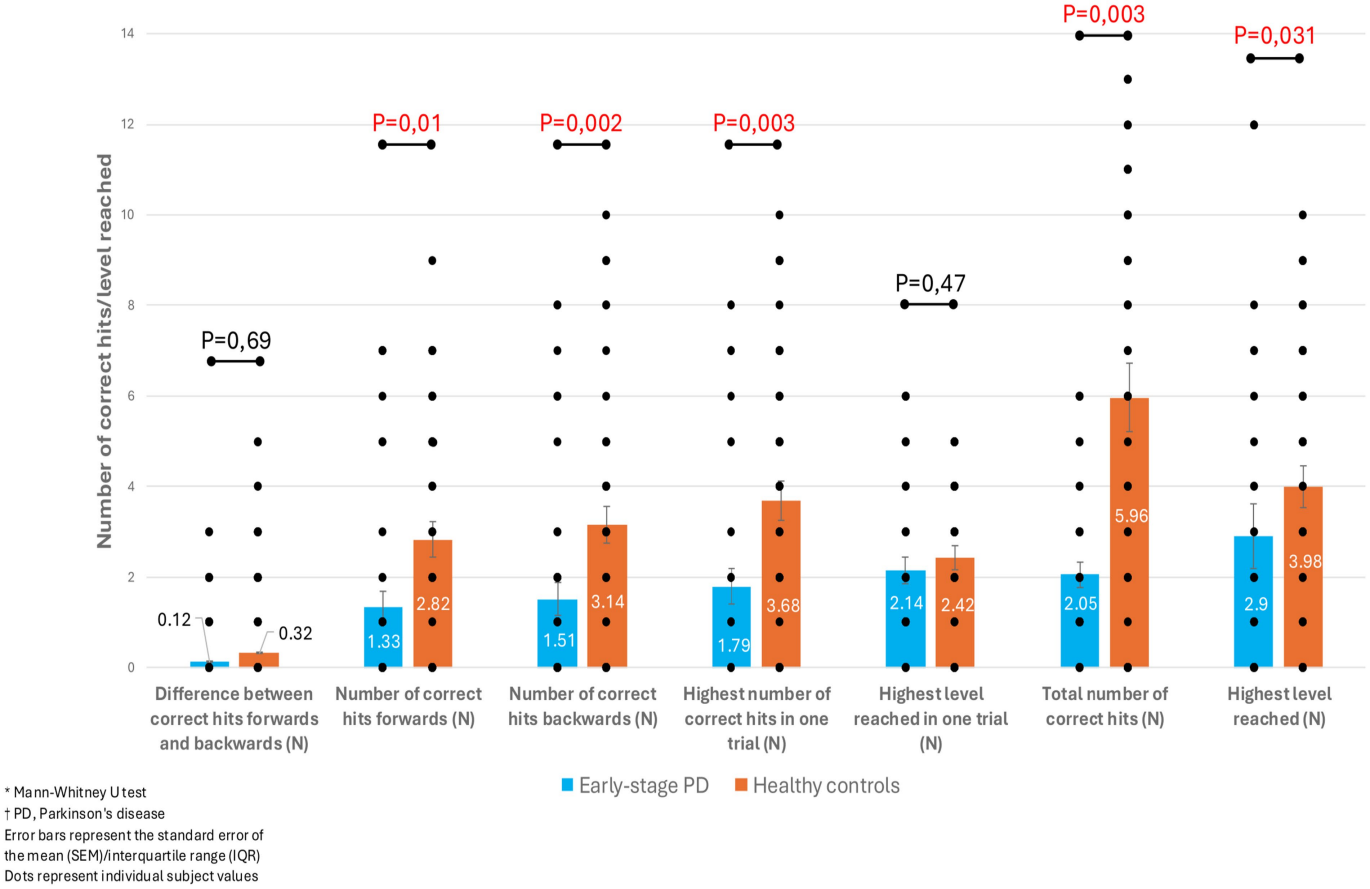
Comparison of parameters of visually guided memory saccades between patients with early-stage PD and healthy controls.

We did not find any statistically significant differences in parameters of reflexive saccades between patients with early PD and healthy controls. On the other hand, patients with PD had significantly worse results regarding visually guided memory saccades. More precisely, they had a lower number of correct hits in both directions, a lower number of correct hits in one trial and overall, and a lower level reached when performing the Corsi test (Figure 2).

When observing antisaccades, patients with early-stage PD had fewer correct hits than healthy controls (6 versus 13, *p* < 0,001, Mann–Whitney *U* test), but there were no differences in other observed parameters between these two groups.

**Figure 3:**
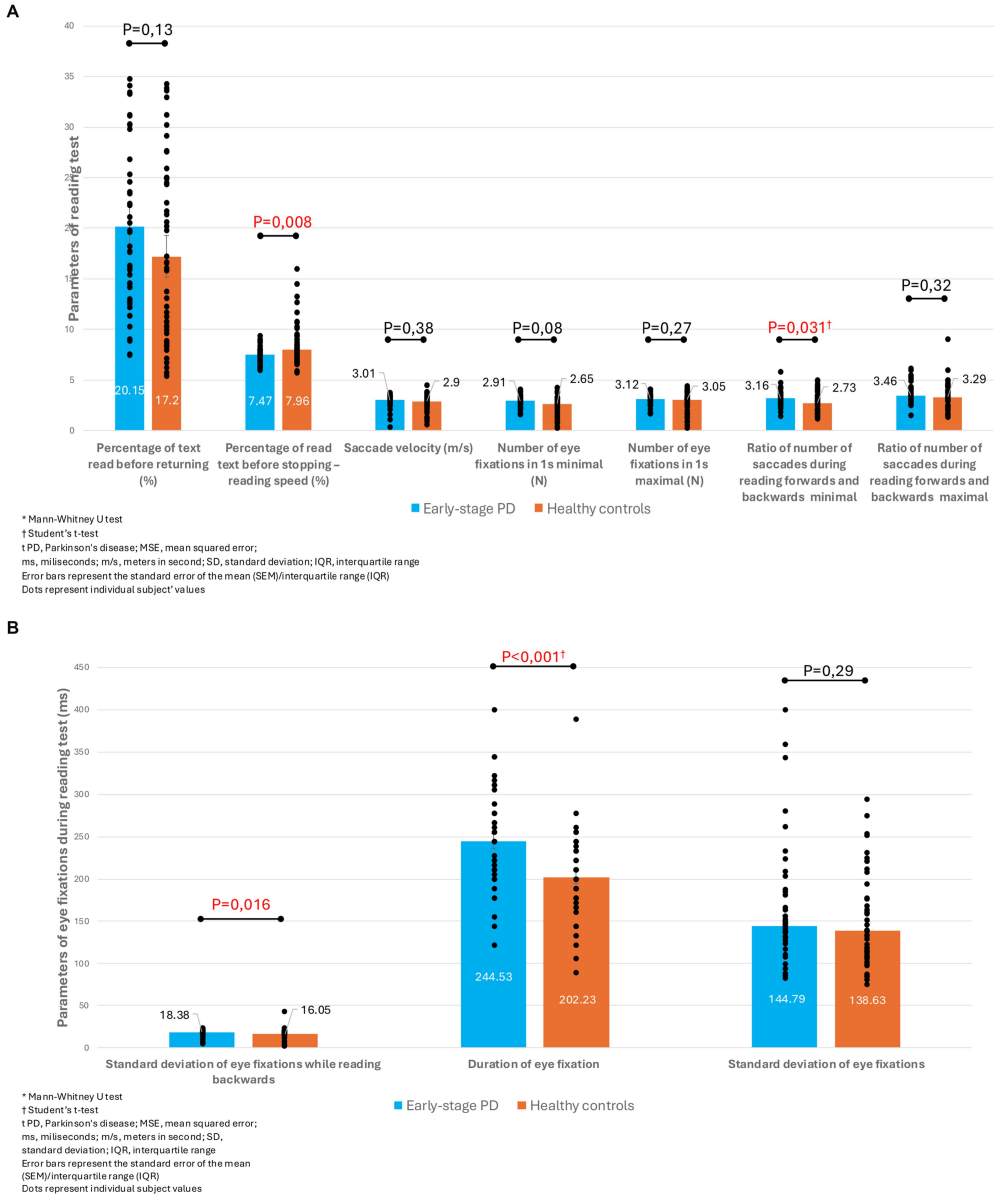
**(A)** Comparison of reading test parameters between patients with early-stage PD and healthy controls. **(B)** Comparison of parameters of eye fixation during reading test between patients with early-stage PD and healthy controls.

During the reading test, patients with early-stage PD showed significantly slower reading speed (evidenced by the percentage of read text before stopping). They also had a larger deviation of duration of eye fixations, a larger minimal ratio of number of saccades forwards and backwards, and a longer average duration of eye fixation (Figures 3A,B).

## 4 Discussion

Our study focuses on early-stage PD patients, which is evidenced by H&Y stage. Our results showed significant impairment of smooth pursuit eye-movements and visually guided memory saccades in patients with early-stage PD. Therefore, it is not surprising that patients with early-stage PD also have problems while reading, with longer duration of eye fixation and slower reading speed. We have not found convincing evidence of impairment of reflexive saccades and antisaccades, which could be explained by their separate neuroanatomical regulation.

Many previous studies (4–6) have shown that PD patients have disorders in various aspects of eye movements. However, there is a paucity of studies that examine the impairment of eye movements in early-stage PD. Also, most studies focus on smooth pursuits and voluntary and reflexive saccades and neglect memory saccades and parameters of reading.

### 4.1 Smooth pursuit movements in early-stage PD

During the clinical examination of patients with PD, the examiner can often notice fragmented smooth movements of the eyeball. However, there is a whole spectrum of other smooth movement disorders that can only be detected using an eye-tracker. It is thought that 67% of patients with PD have some impairment of smooth tracking movements, which is the highest prevalence of all possible types of ocular movements (4). It is evident that patients with PD have significantly worse smooth pursuit velocities compared to healthy controls and that these changes are especially present during execution of faster moves. Additionally, patients with PD have more frequent saccadic intrusions, which can manifest as fixations and thus be quantified (24). It is not entirely clear at which stage of the disease these changes occur, although in the study by Bares et al. (25) damage to movement velocity was already present after 2.2 years of disease duration. In addition, earlier studies also looked at phase of movement but did not provide consistent data on whether this parameter is also impaired (24). In our study, we confirmed that subjects with early-stage PD have a slower velocity of smooth pursuit movement. Also, our subjects with PD also had a longer phase and a smaller range of motion of smooth pursuit movement, which are expected characteristics in patients suffering from PD. The mentioned parameters indicated already significantly impaired smooth movements because they are present in all observed cycles and in both the horizontal and vertical axes. In the fastest movement cycle in PD patients, movement accuracy was also impaired, but only in the vertical axis. However, we did not find significant changes in the number of fixations. These results could indicate that saccadic intrusions occur in later stages of PD, after the velocity, phase, and range of movements have already been damaged.

In regulation of smooth eye movements, signals from different parts of the cortex affect the frontal and parietal eye fields (FEF and PEF, respectively), which are the initiators of movement and act on the superior colliculus to execute the movement. The outer and inner parts of the globus pallidus serve to fine-tune these movements (26). Even though other centres provide the signal for smooth eye movements, the globus pallidus plays a crucial role in fine-tuning these movements and determining their execution accuracy, directly impacting the FEF. In addition, the globus pallidus also participates in a direct and indirect pathway, acting on the modification of motor skills. Due to the destruction of the SNc, the dopaminergic innervation of the striatum is reduced, which is why we have a reduced inhibition of the otherwise inhibitory activity of the globus pallidus, which leads to a reduced modification of movements, along with their slowing down and blocking. The signal is amplified (due to the convergence of projections of the basal ganglia towards the thalamus), which is why changes in smooth tracking movements are already evident with less dopaminergic denervation in the earlier stages of the disease.

### 4.2 Reflexive saccades in early-stage PD

It is known that reflexive saccades in PD patients are hypometric and of extended latency, with a greater number of errors during execution (27). Some studies have found these changes in *de novo* PD patients and those with early-stage PD (13, 17), while others say that reflexive saccades become impaired only later (6). We also did not find any changes in reflexive saccades, which suggests that they might be initially preserved until later stages of disease. The rationale for this hypothesis is the distinct neuroanatomical regulation of reflexive saccades. PEF has direct projection to the upper colliculus and initiates these types of saccades, bypassing the basal ganglia (28). We believe that this mechanism is a possible explanation for the preservation of these components in our subjects with early-stage PD.

### 4.3 Memory saccades in early-stage PD

In contrast to reflexive saccades, patients with early-stage PD in our study had a significantly higher number of errors during the performance of memory saccades than healthy controls, regardless of whether the tasks are performed backwards or forwards. These respondents also demonstrate a lower number of correct hits, both in a single trial and overall, as well as a lower overall performance level. We know that memory saccades are a subtype of voluntary saccades, whose generator is also the FEF, which projects directly to the SC. The signal from previous visual stimuli is stored in the cortical–subcortical oculomotor loop (in which numerous memory storage structures participate, i.e., the dorsolateral prefrontal cortex but also the nucleus caudatus and SNr). The FEF is then activated by this signal and makes memory-guided saccades with a projection to the SC (29). We previously mentioned that changes in the FEF and the dorsolateral prefrontal cortex (13), which affect executive functions and memory, are thought to be responsible for disturbances in memory saccades. Considering that there were no significant differences between our groups of subjects in cognitive function testing, we did not expect that changes would manifest themselves in memory saccades. It is possible that the initial degeneration of basal ganglia leads to dysfunction in the storage of visual stimuli, implying that these saccades serve as a sensitive indicator of subtle cognitive changes, even in the early stages of PD.

### 4.4 Antisaccades in early-stage PD

The prefrontal cortex is also important in the pathophysiology of antisaccades, which cause the suppression of reflex saccades and enable looking in the opposite direction. Therefore, it is not surprising that cognitive impairments also play a major role in lengthening latencies and increasing the number of performance errors (10). Our subjects with PD showed a higher number of errors when performing saccades, but without changes in other parameters. The findings are in favor of the initial changes, with the fact that we cannot ignore the possibility that the changes would be more evident in a larger number of subjects and in patients with longer durations of PD.

### 4.5 Reading in early-stage PD

When we look at the bigger picture and the integration of different bulbomotor parameters, it is necessary to analyse the reading test. In addition to the standard previously mentioned parameters, with this test we can also evaluate the duration of fixation and the standard range of fixation (range between different fixation durations). It is generally considered that patients with PD are more easily distracted due to reduced ability to concentrate, which is presented by breaking fixation and a larger standard deviation of fixation. This instability of fixation is a marker of neurodegenerative diseases and results in poorer quality of reading. Additionally, due to difficulty in reading, they can have longer average fixation durations and more frequent saccadic movements during reading (30). This results from damage to the neural circuits between the basal ganglia and the dorsal prefrontal cortex, as well as between the basal ganglia and the upper colliculus (7). In our study as well, subjects with early-stage PD showed a longer duration of fixations and a greater range between individual fixations when reading backwards. They also had a lower percentage of the screen read at one time before returning to the already read part of the text, which highlights their impaired reading speed. Thus, we also proved the existence of fixation instability in our subjects, which is characteristic of PD. Unlike other studies, we did not find an increased number of saccades during reading, possibly because of the still early stage of the disease, when saccadic intrusions (and especially square wave jerks) are not so present.

Evident differences in speed of smooth pursuit eye movements between patients with PD and healthy controls raise questions for possible utilization of eye-tracker in non-invasive diagnosis of PD. Of course, in order to implement the mentioned method, additional tests are needed that would confirm our results, with the introduction of a standardized method and a battery of tests that would be used in the examination of eye movements.

### 4.6 Study limitations

Our research was a cross-sectional study. However, the number of patients included in the study and the number of healthy controls is bigger compared to most of the studies done so far. Furthermore, our subjects with PD were not *de novo* patients and had already received antiparkinsonian therapy. Some studies suggest that antiparkinsonian therapy can influence the eye-movement parameters in PD. However, the LEDD was rather low suggesting that the dopaminergic medication might not have influenced our results considerably.

## 5 Conclusion

Impaired smooth pursuit movements, memory-guided saccades and reading functions are present in early-stage PD, even without other expressed motor symptoms. The lack of direct pathway signalling through the globus pallidus and degeneration of oculomotor loop structures, important for storage of visual stimuli, are possible pathophysiological explanations. On the other hand, reflexive saccades and antisaccades seem to be preserved in these patients. These findings could potentially contribute to the development of new and non-invasive diagnostic biomarkers in PD.

## Data Availability

The datasets presented in this article are not readily available because no restrictions. Requests to access the datasets should be directed to zvonepop@gmail.com.
