# Supplementary material for: Smooth pursuit and memory saccades are impaired in early-stage Parkinson’s disease patients
Source: Front Neurol. 2026 Jan 22;16:1702050. doi: 10.3389/fneur.2025.1702050 (PMC12873708; doi:10.3389/fneur.2025.1702050)

Figure 1.

Exclusion criteria
Structural brain damage
Uncorrected visual impairment
Dementia (MoCA<18)

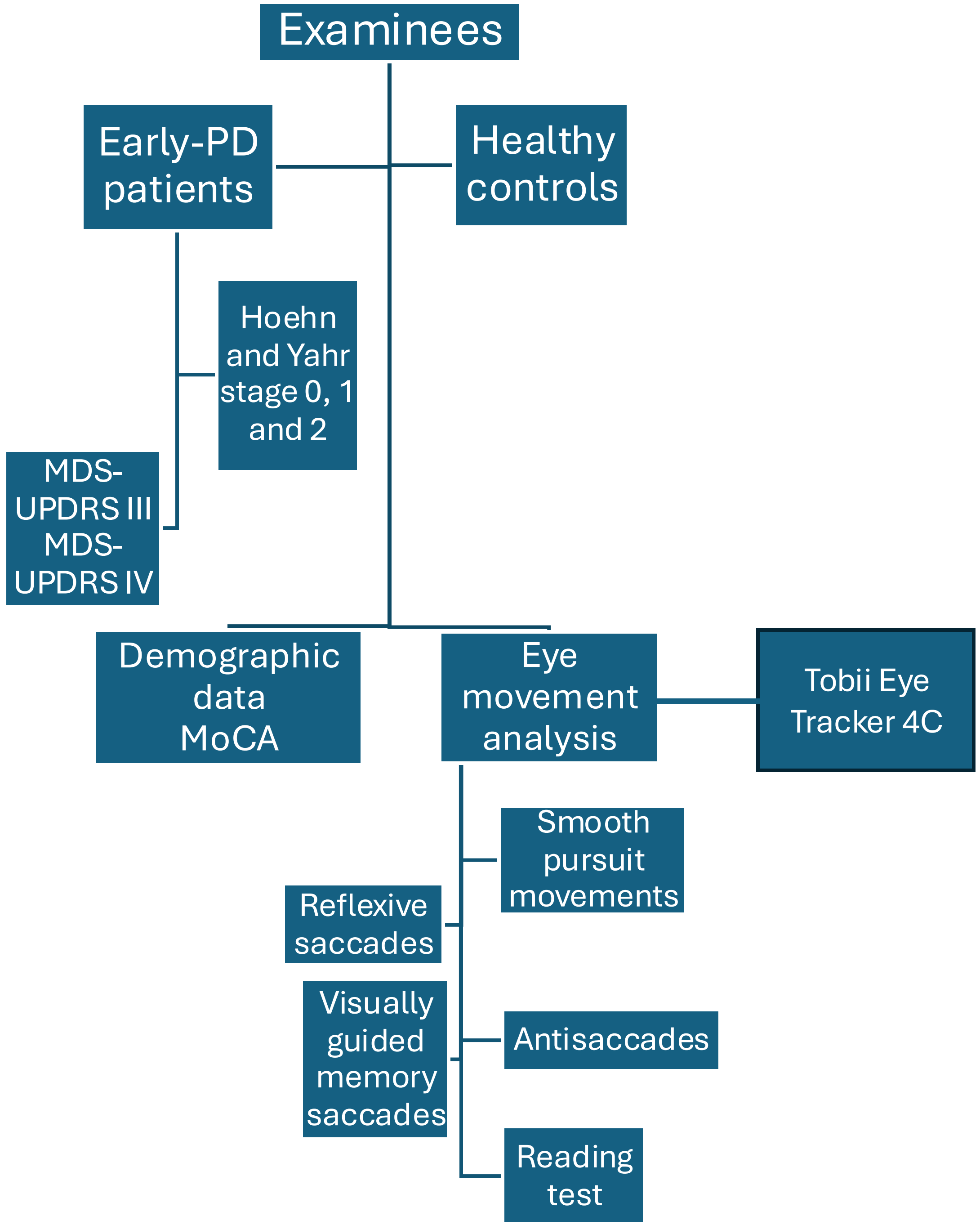

Supplement: Supplementary file 1 [file Data_Sheet_1.pdf]
