# Supplementary figures and images for: Smooth pursuit and memory saccades are impaired in early-stage Parkinson’s disease patients
Source: Front Neurol. 2026 Jan 22;16:1702050. doi: 10.3389/fneur.2025.1702050 (PMC12873708; doi:10.3389/fneur.2025.1702050)

Figure 2A.

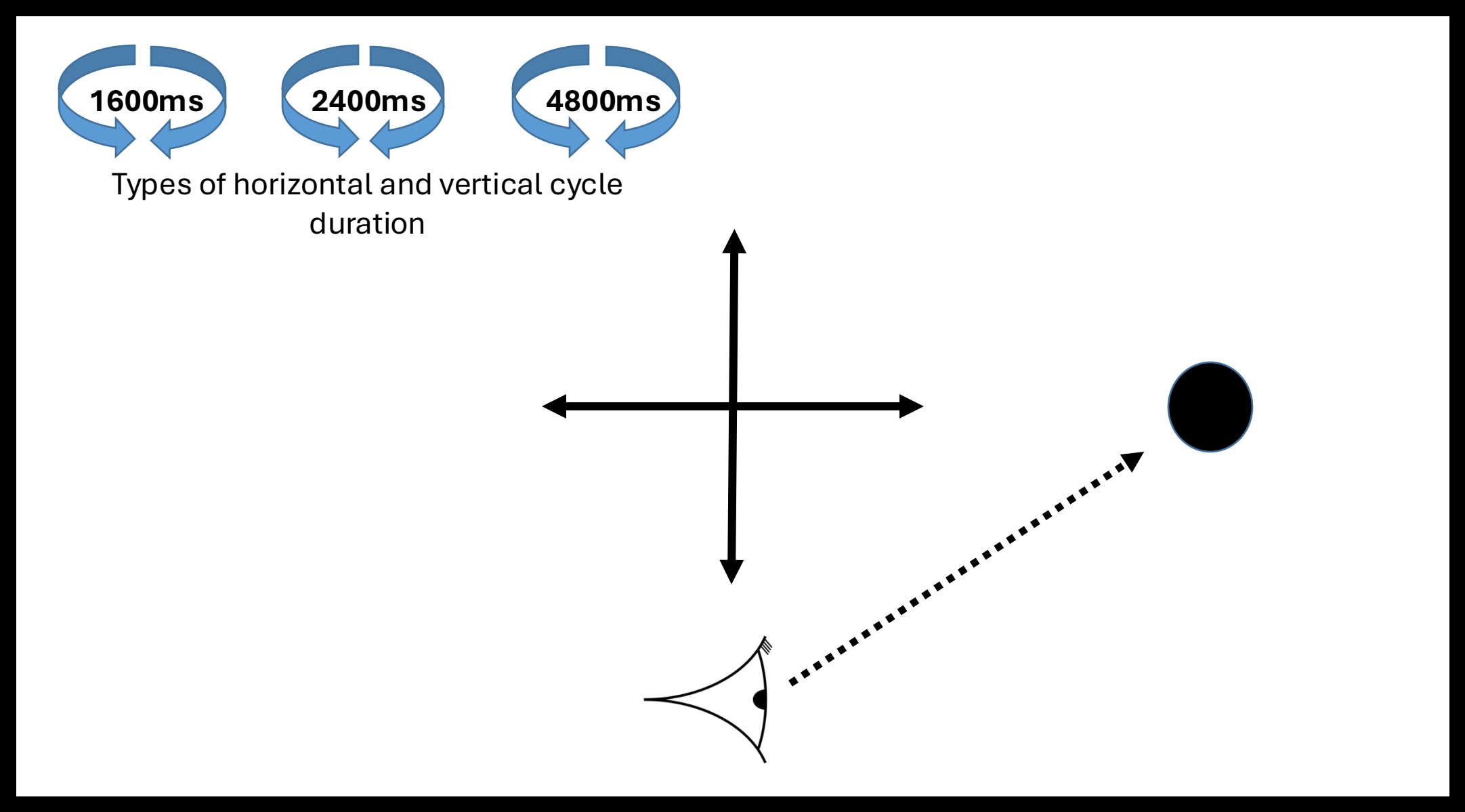

Figure 2B.

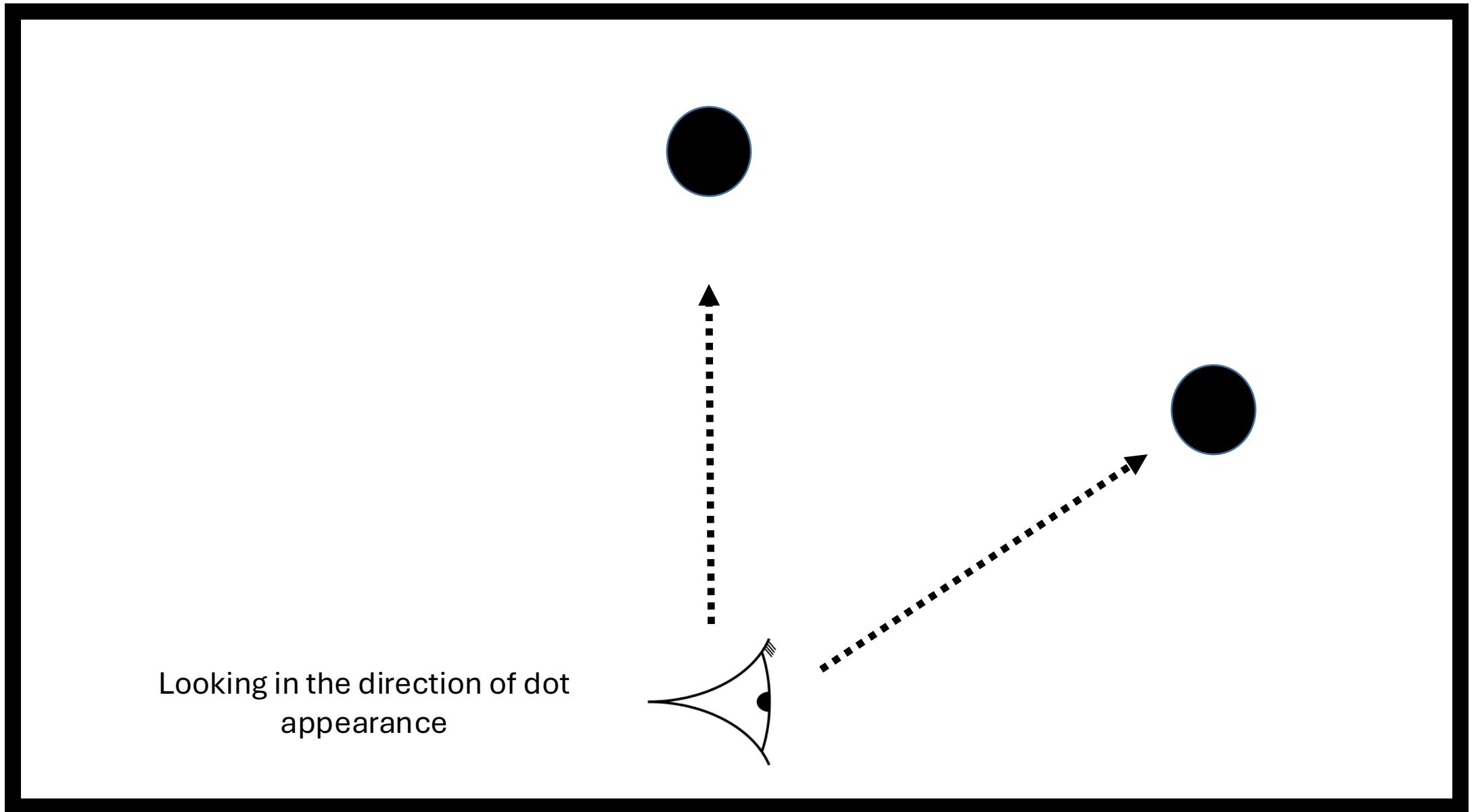

Figure 2C.

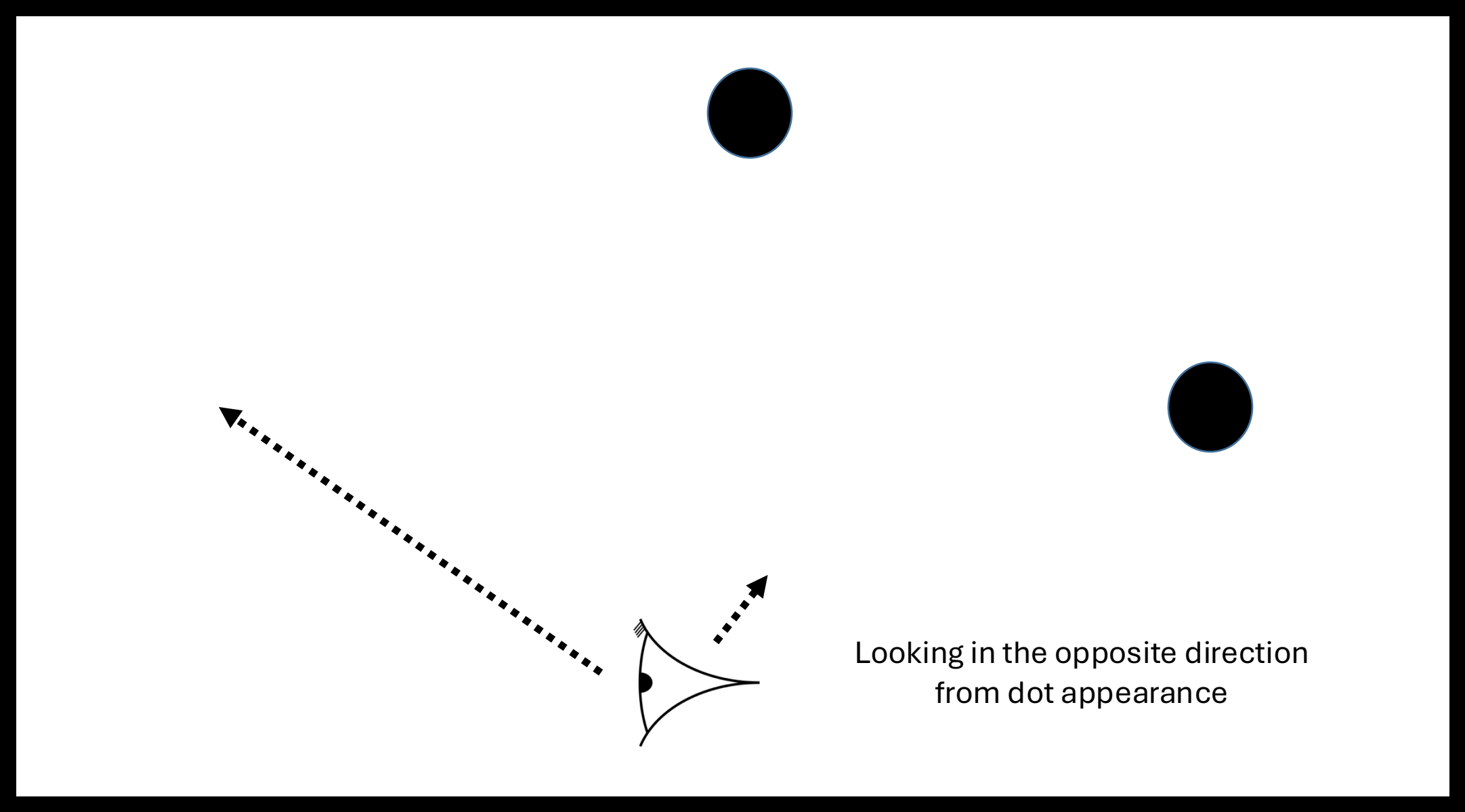

Figure 2D.

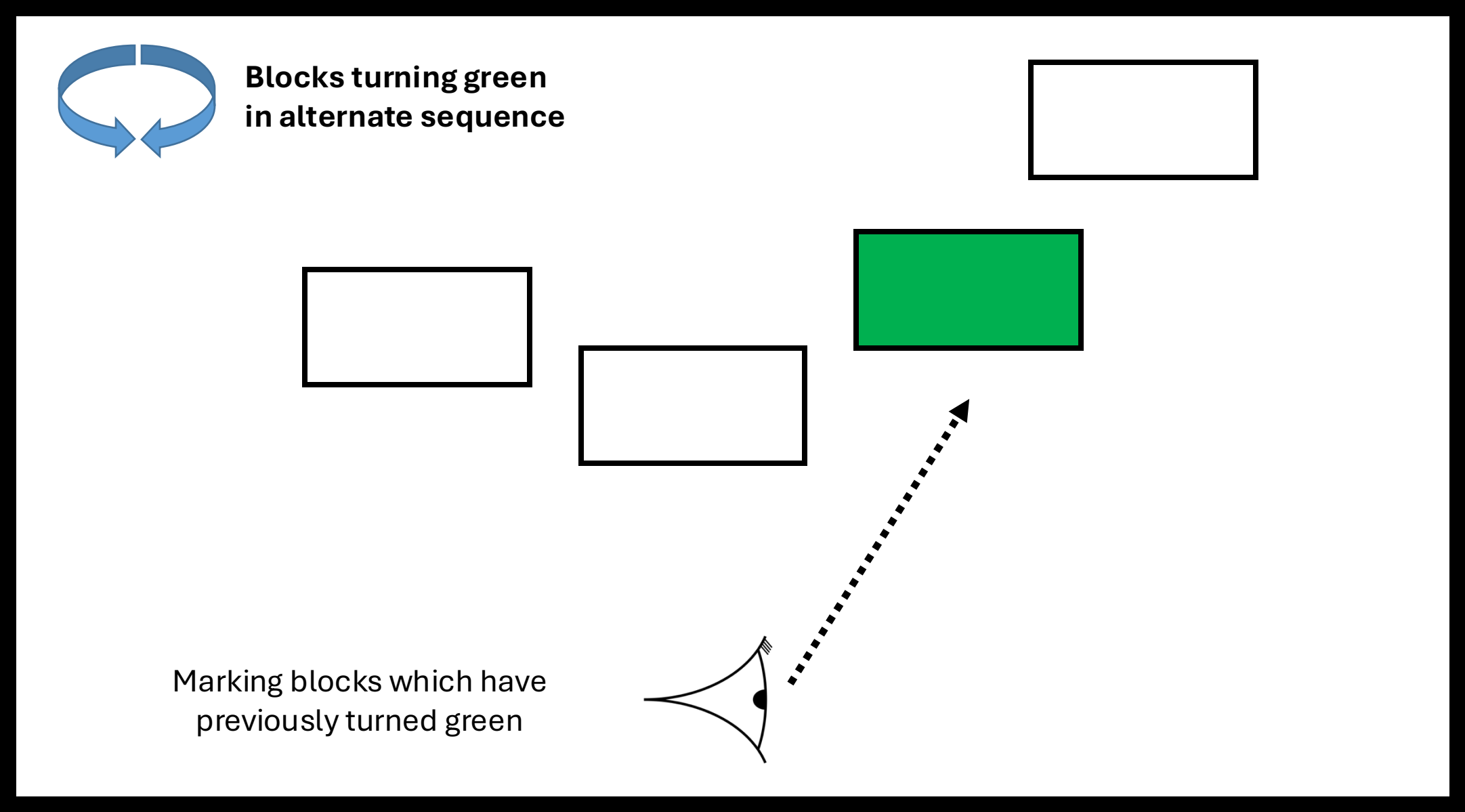

Supplement: Supplementary file 2 [file Data_Sheet_2.pdf]
